# Identifying the most effective behavioural assays and predator cues for quantifying anti-predator responses in mammals: a systematic review

**DOI:** 10.1186/s13750-023-00299-x

**Published:** 2023-04-01

**Authors:** Natasha D. Harrison, Rochelle Steven, Ben L. Phillips, Jan M. Hemmi, Adrian F. Wayne, Nicola J. Mitchell

**Affiliations:** 1 School of Biological Sciences, University of Western Australia, Crawley, WA 6009 Australia; 2 Environmental and Conservation Sciences, Harry Butler Institute, Murdoch University, Murdoch, WA 6150 Australia; 3 School of BioSciences, University of Melbourne, Parkville, VIC 3010 Australia; 4 The UWA Oceans Institute, The University of Western Australia, Perth, WA 6009 Australia; 5 Biodiversity and Conservation Science, Department of Biodiversity, Conservation and Attractions, Manjimup, WA 6258 Australia

**Keywords:** Anti-predator behaviour, Behavioural adaptation, Behavioural assay, Effect size, Evidence synthesis, Predator avoidance, Predator cue, Prey naïveté

## Abstract

**Background:**

Mammals, globally, are facing population declines. Protecting and breeding threatened populations inside predator-free havens and translocating them back to the wild is commonly viewed as a solution. These approaches can expose predator-naïve animals to predators they have never encountered and as a result, many conservation projects have failed due to the predation of individuals that lacked appropriate anti-predator responses. Hence, robust ways to measure anti-predator responses are urgently needed to help identify naïve populations at risk, to select appropriate animals for translocation, and to monitor managed populations for changes in anti-predator traits. Here, we undertake a systematic review that collates existing behavioural assays of anti-predator responses and identifies assay types and predator cues that provoke the greatest behavioural responses.

**Methods:**

We retrieved articles from academic bibliographic databases and grey literature sources (such as government and conservation management reports), using a Boolean search string. Each article was screened against eligibility criteria determined using the PICO (Population–Intervention–Comparator–Outcome) framework. Using data extracted from each article, we mapped all known behavioural assays for quantifying anti-predator responses in mammals and examined the context in which each assay has been implemented (e.g., species tested, predator cue characteristics). Finally, with mixed effects modelling, we determined which of these assays and predator cue types elicit the greatest behavioural responses based on standardised difference in response between treatment and control groups.

**Review findings:**

We reviewed 5168 articles, 211 of which were eligible, constituting 1016 studies on 126 mammal species, a quarter of which are threatened by invasive species. We identified six major types of behavioural assays: behavioural focals, capture probability, feeding station, flight initiation distance, giving-up density, and stimulus presentations. Across studies, there were five primary behaviours measured: activity, escape, exploration, foraging, and vigilance. These behaviours yielded similar effect sizes across studies. With regard to study design, however, studies that used natural olfactory cues tended to report larger effect sizes than those that used artificial cues. Effect sizes were larger in studies that analysed sexes individually, rather than combining males and females. Studies that used ‘blank’ control treatments (the absence of a stimulus) rather than a treatment with a control stimulus had higher effect sizes. Although many studies involved repeat measures of known individuals, only 15.4% of these used their data to calculate measures of individual repeatability.

**Conclusions:**

Our review highlights important aspects of experimental design and reporting that should be considered. Where possible, studies of anti-predator behaviour should use appropriate control treatments, analyse males and females separately, and choose organic predator cues. Studies should also look to report the individual repeatability of behavioural traits, and to correctly identify measures of uncertainty (error bars). The review highlights robust methodology, reveals promising techniques on which to focus future assay development, and collates relevant information for conservation managers.

**Supplementary Information:**

The online version contains supplementary material available at 10.1186/s13750-023-00299-x.

## Background

### The need to quantify anti-predator responses

Mammal populations are experiencing alarming rates of extinction [1–3] due to anthropogenic impacts such as habitat loss and fragmentation, illegal hunting, and exotic predators [4]. Redressing this loss of biodiversity requires well-informed and well-tested management interventions. Many of these interventions will need to be underpinned by a mechanistic understanding of species’ behaviour.

How an animal responds to predators has substantial bearing on its ability to survive. Predation, particularly from introduced predators, has been a major driver of mammal declines and extinctions around the world [5–9]. This is especially true for individuals and populations that have had limited or no exposure to predators, such as many island populations [10, 11], individuals raised in captivity, and those moved to an environment with novel predators [12–14]. Improving our understanding of how animals behave in response to predatory stimuli should provide crucial insights for their conservation management and can improve our ability to retain antipredator traits in managed populations [12, 15, 16]. An animal’s response to predators may be either behavioural (e.g. spatial and temporal avoidance [17, 18], avoiding detection [19] and evasion [20]), or chemical [21] and physical defences [22]. Behavioural responses are likely to be more plastic and responsive at shorter time frames than physical responses and are therefore particularly important when considering the acute impacts of predators on the persistence of predator-naïve species. Such behavioural responses may be closely linked to animal personalities (or behavioural syndromes)—repeatable inter-individual differences in a set of behaviours [23], traditionally assessed along a bold-shy continuum [24, 25]. Personality is important to consider in the context of ecology as it contributes to inter- and intra-individual variations in behaviour, influencing both the ability of an individual to respond to environmental changes, as well as the efficiency with which natural selection can act [23].

Animal behaviour can be influenced by human interactions. For example, the coexistence of humans and wildlife in urban areas often selects for boldness in individuals [24, 25]. Conservation interventions can also indirectly affect behavioural responses in target species. Common strategies employed to prevent faunal extinctions include captive breeding [26], translocations (the deliberate movement of animals from one population or site for release in another [27]), and establishment of populations in predator-free havens (areas isolated from predators through a geographical or physical barrier, such as islands or fenced enclosures [28–30]). Such approaches have secured a number of populations of mammals, including African elephants [31, 32], European lynx [33], elk [34], giant pandas [35], and Tasmanian devils [36]. Despite their initial successes, these strategies are at risk of longer-term failure if they select for bold, predator-naïve behaviours. Such haven-adapted populations would be particularly vulnerable to acute population collapses from predator incursions and would face substantial challenges when reintroduced to areas containing predators.

Australia provides a compelling case study to illustrate the challenges of mammal conservation. More than one third of modern mammal extinctions have occurred in Australia, largely due to the introduction of feral cats and foxes [37]. In response, havens free of introduced predators are a key component of conserving much of the remaining mammal fauna [29, 30, 38]. Australia’s current network of havens provides habitats for at least 32 mammal species, and has secured at least 188 populations and sub-populations [29]. Evidence is emerging, however, that in the absence of feral and/or native predators, havened populations no longer exhibit anti-predator behaviours [13, 39–44]. This renders individuals in these populations fundamentally unfit for reintroduction back into where predators still persist. Because the success of many translocations has ultimately been compromised by predation [38, 45, 46], the future of mammal conservation in Australia, and more broadly, hinges on developing methods and strategies that can quantify and conserve antipredator behaviours in havened and translocated populations [42].

To undertake an adaptive management approach, we require monitoring and evaluation of anti-predator responses in mammalian species. Despite awareness that behavioural traits such as boldness or shyness can influence conservation outcomes, measuring such traits is rarely incorporated into monitoring and management [16, 47]. Anti-predator responses have only recently been identified as a potential barrier to the success of conservation projects [13, 40–42], and while an array of academic literature exists that details various methods for measuring these behaviours [15, 41, 42, 48–53], accessing the methodologies, comparing them for rigor, and identifying the most appropriate measure is labour intensive. Stakeholders, such as conservation and population managers, are likely to be seeking this information, but are also likely to be limited by the time and resources necessary to find it. Ultimately, we currently lack a robust framework for the universal monitoring and evaluation of anti-predator traits [54]. The first step to developing such a framework is to understand which behavioural assays have been used, which are the most effective (capture or provoke the greatest behavioural response), and whether the type of predator cue is important. In the absence of this crucial information, the adoption of inappropriate and poorly-performing behavioural metrics may prevail.

### Identification and engagement of stakeholders

**Fig. 1:**
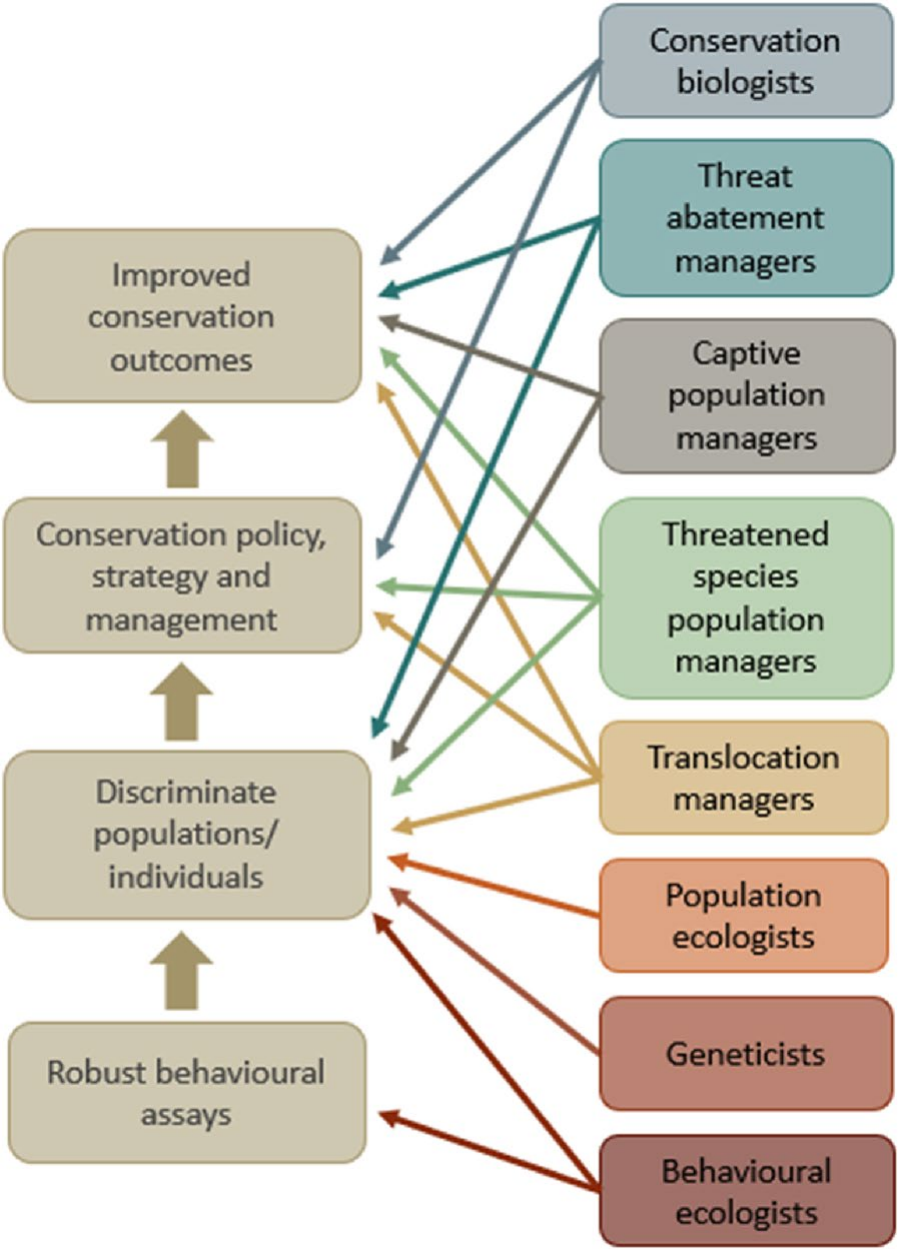
End-user stakeholder groups (right-hand boxes) consulted when designing a systematic review of methods that quantify anti-predator behaviour in mammals. Arrows indicate each groups’ broad interests in the various steps (left-hand boxes) required for improving conservation outcomes. Robust behavioural assays facilitate the accurate discrimination of individuals or populations based on certain desirable behavioural attributes. This information can then be used to directly inform conservation management, whereby effective conservation management strategies ultimately lead to improved conservation outcomes

In addition to the review team, stakeholders relevant to this review have been identified as those who research or manage animal populations, for example, members of species recovery teams (Fig. 1). To ensure the information collected throughout this review is tailored toward the target audience, and thus of the most relevance for application, a variety of stakeholders from each of the categories in Fig. 1 were consulted during the development of the protocol. We invited 27 stakeholders to comment on the draft protocol, and after receiving 16 replies (ten from Australia and six from other countries), we incorporated their suggestions.

## Objective of the review

We present all available behavioural assays for measuring or quantifying anti-predator responses in mammals by collating information into an accessible format. Specifically, we: (1) reveal different methods, (2) describe the context within which each method was conducted, and (3) highlight methods or aspects that warrant further examination, thus guiding the future development of behavioural assays. Further, using a modelling approach, we then identify which types of behavioural assays and predator cues elicit the greatest responses in mammals (difference in effect size between the treatment and control conditions). A formal evidence synthesis is required to explore all potential methods and to avoid bias toward those published in academic journals, because much information may come from governmental reports and species recovery plans [16, 55]. The final review acts as a guide: it highlights existing methodologies and provides additional information to assess their relevance, allowing stakeholders to easily select the most appropriate and effective behavioural assay for their purpose.

Using the PICO (Population–Intervention–Comparator–Outcome) framework [56], we have broken our review into two questions that will define our search scope. We will first systematically map all known methodologies answering a primary question: *what behavioural assays have been used to quantify anti-predator responses in mammals?* The elements of this question are:

**Population**: Free-living, wild-caught, or captive mammals (global)
**Intervention**: (i) a behavioural assay that quantifies anti-predator responses to predator exposure
  (ii) a behavioural assay that quantifies anti-predator responses to predator cues

Articles that conform to both the **Population** and **Intervention** criteria will be used to answer this primary question. A secondary question we seek to answer will be assessed quantitatively by modelling the data collected from each article, asking: *which assay-types and predator cues elicit the greatest behavioural responses*? This question utilises the same **Population** and **Intervention** criteria as the primary question but requires further assessment using **Comparator** and **Outcome** criteria to select studies for the systematic review. The additional elements of the secondary question are:

**Comparator**: Comparison between levels of predator exposure (e.g., before versus after exposure, exposure versus no exposure) or comparison between exposure to a predator cue versus a control.
**Outcome**: Difference in the behavioural response between the treatment (e.g., predator/predator cue exposure) and control conditions. Metrics of responses will differ between studies depending on assay type and will be compared using standardised effect sizes.

**Fig. 2:**
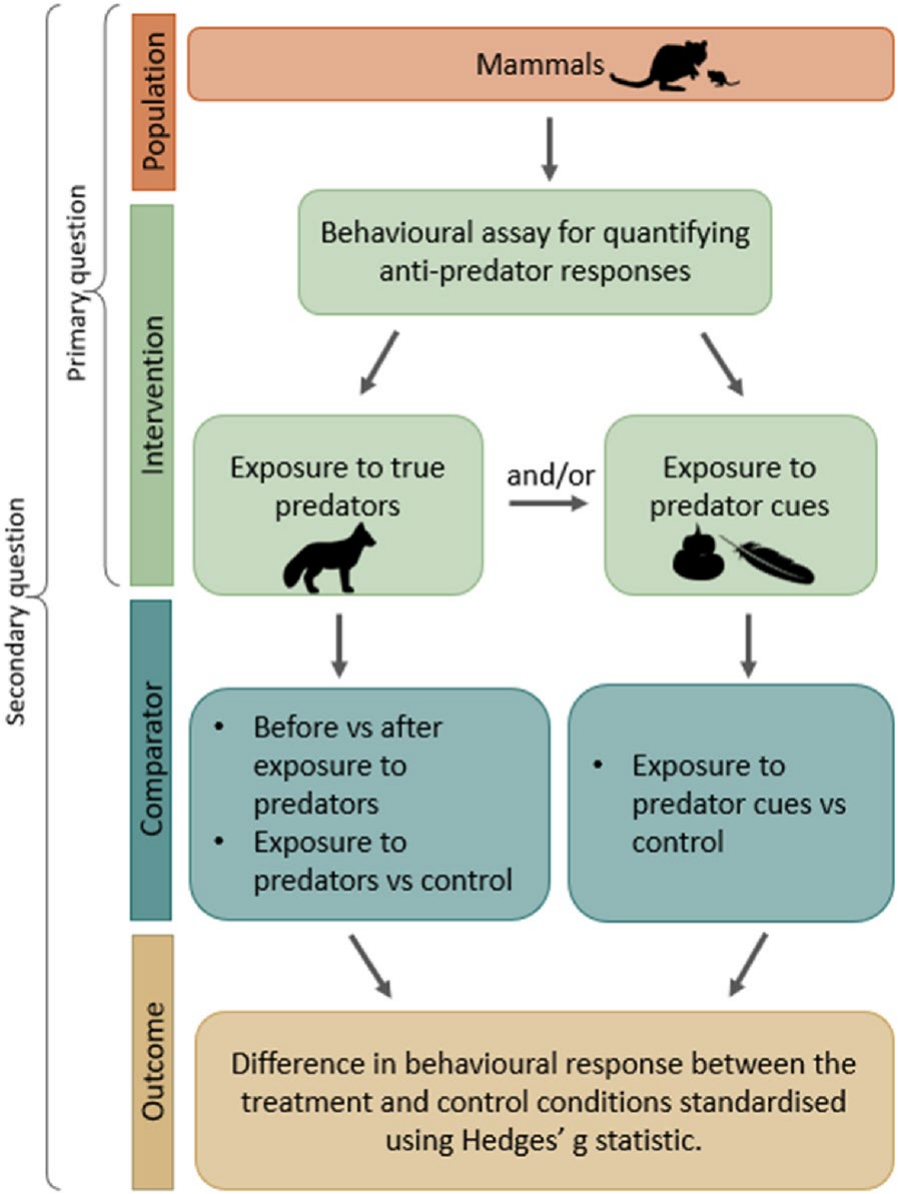
Elements of target questions illustrated using the PICO framework

Articles that involve at least one **Comparator** element can then additionally be considered for the systematic review to investigate which **Intervention** elements (behavioural assays and predator cues) produce the greatest **Outcome**. The PICO elements of our two questions are illustrated in Fig. 2.

## Methods

This systematic review follows a pre-defined protocol [57] that conforms to the CEE guidelines [56] and ROSES reporting standards (Additional file 1, ROSES checklist).

### Deviations from the protocol

We were unable to use the entire search string to search *EThOS: UK Theses and Dissertations*, instead we searched using the search terms “anti-predator” and “antipredator”. The website https://opengrey.eu could not be searched as it has been removed. One eligibility criterion was added for the screening process; the article must contain primary results (e.g. a meta-analysis or book chapter that presents and cites findings from another study will not be included) as articles that present results from other studies do not contain enough methodological detail for this review, and we are confident that relevant original studies will be captured by our search strategy. We also clarified that to satisfy the population criteria, individuals must not have been physically or physiologically altered (e.g., had any substance administered or limb removed) as this may affect their response and bias our quantitative component. We intended to use the CEE Critical Appraisal tool to evaluate potential biases from our studies, however, found that many of the questions lacked relevance for studies of animal populations (as they were directed toward human subjects). We refined the criteria to better suit our review (Additional file 3).

### Search for articles

#### Search terms and strings

To develop a search strategy, an initial scoping exercise was conducted using a test-list of 10 benchmark articles that assess anti-predator responses (Additional file 2), each selected by the review team as they cover a variety of different assays and predator scenarios. The titles, key words, and abstracts of each scoping article were mined, both manually, and using word clouds (R package *wordcloud* [58]; in the R environment [59]), to determine the most appropriate search terms [60]. An initial search string was then created using Boolean operators to combine the relevant terms based on the review team’s knowledge, and the terms identified from the scoping articles. Trial searches were conducted using the *Web of Science: Core Collection*. We systematically removed terms that appeared to broaden the search outside the scope of the review. To ensure the proposed strategy adequately returned relevant literature, the search output was scanned for relevant articles and each of the scoping benchmark articles. Unreturned articles were then closely inspected, and the search strategy was adjusted until it retrieved all 10 benchmark articles [56]. The comprehensiveness of the search strategy was then tested using a list of 5 independent articles (Additional file 2), all of which were retrieved by the final search strategy. The final search string (modified for each specific database language) was: TS = ((("antipredator response$" OR "anti-predator response$" OR "antipredator behavio$r" OR "anti-predator behavio$r" OR "escape behavio$r" OR "giving$up density" OR "FID" OR "GUD" OR "flight initiation distance") AND ("predator exposure" OR "prey naïveté" OR "naïve prey" OR "los$" OR "trait" OR "predator avoid*")) OR (("predator recognition" OR "predator exposure" OR "predation risk" OR "introduced predator$" OR "novel predator$" OR "predator odour") AND ("naïve prey" OR "prey naïveté" or "escape behavio$r" OR "giving$up density" OR "flight initiation distance" OR "FID" OR "GUD" OR "predator odour")) OR (("antipredator response$" OR "anti-predator response$" OR "antipredator behavio$r"OR "anti-predator behavio$r" OR "escape behavio$r") AND ("predator recognition" OR "predator exposure" OR "introduced predator$" OR "novel predator$")))

#### Search limitations

All searches were conducted in English which may exclude studies from regions that do not predominantly speak English. Searches were conducted between 7th and 21st December 2021.

### Search sources

#### Academic literature

Using the above search string, we searched the following bibliographic databases from which to collect peer-reviewed journal articles: *Web of Science (Core Collection, BIOSIS Citation Index, Zoological Record, CAB abstracts)* and *Scopus*.

#### Grey literature

To reduce bias toward published literature, we also searched a variety of grey literature sources [54, 55]. We collated theses and dissertations from two bibliographic databases specific to grey literature: *Proquest Dissertation* (using the above search string) and *EThOS: UK Theses and Dissertations* (using the search terms “anti-predator” and “antipredator”). Conference proceedings were searched in the *Web of Science* database using the predetermined search string. The website trove.nla.gov.au was also searched, using the search terms “anti-predator” and “antipredator”. Specialist documents were searched for from within the following repositories, using the search terms “anti-predator” and “antipredator”: IUCN general publications (https://portals.iucn.org/library/dir/publications-list); IUCN Conservation Planning Specialist Group (http://www.cpsg.org/document-repository); Conservation Evidence (http://www.ConservationEvidence.com); WWF (https://www.worldwildlife.org/publications). A web-based search engine, Google (www.google.com), was used to supplement our search results. The first 50 links returned using each combination of the search terms “anti-predator/antipredator” and “behaviour/behavior”, were inspected and added to the article pool if not yet identified [61].

### Article screening and study eligibility criteria

#### Screening process

Articles were uploaded into CADIMA software [62], where duplicates were removed and article screening took place. To remove bias, two screeners independently reviewed articles at title and abstract level simultaneously, followed by the full text versions, to decide which met the inclusion criteria. Each screener assessed an overlap of 50 articles at both the title/abstract stage, and at the full text stage (1.5% and 9.5% respectively). Reliability between screeners was assessed using Kappa calculations and were deemed reliable (inter-screener reliability scores of 1 and 0.8 at title/abstract and full text stages respectively). In instances where screeners did not agree on the inclusion/exclusion of an article, they discussed, and consulted a third member of the review team if a decision could not be reached. If reviewers found themselves assessing their own work, a third impartial member of the review team supervised the assessment of any conflicting articles. A full list of excluded articles, detailing reasoning for their exclusion can be found in Additional file 4.

### Eligibility criteria

Each article was screened against eligibility criteria based on the PICO framework as outlined in Table 1.

**Table 1:** Study eligibility criteria based on PICO (Population–Intervention–Comparator–Outcome) framework

Population	Eligible subjects include any population of non-human terrestrial mammals (free-living, wild-caught, captive, or domesticated) from around the world. We do not include studies that have used simulated populations. Individuals must not have been physically or physiologically altered
Intervention	Eligible studies use behavioural assays to quantify anti-predator behaviour in response to: (i) Exposure to live true predators (ii) Exposure to predator-related cues, or events that represent a proxy for predatory situations (studies with humans as the predator can be included)
Comparator	The study must contain at least one of the following comparisons [12]: (i) A before/after comparison (BA) that investigates how anti-predator responses change before and after exposure to predators (ii) A control/intervention comparison (CI) that compares anti-predator responses between a group exposed to the predator/s and a designated control group not exposed (iii) A control/intervention comparison (CI) that compares anti-predator responses of individuals exposed to both a predator cue and a control treatment (iv) A before/after/control/intervention comparison (BACI) combining the above components
Outcome	Metrics for behavioural responses vary between assays and are be compared using standardised effect sizes (the difference in mean behavioural responses between the treatment and control conditions). To calculate standardized effect sizes (using Hedges’ g [63]), articles must provide (i) the mean response to each treatment, (ii) its corresponding variance (standard deviation, standard error or variance), and (iii) the sample size for each treatment
Other	The article must contain primary results (e.g. a meta-analysis or book chapter that presents and cites findings from another study will not be included)

Articles that satisfied the **Population** and **Intervention** eligibility criteria were used to pursue the primary question, and were then additionally assessed against the **Comparator** and **Outcome** eligibility criteria for inclusion in the secondary quantitative component where they addressed the effectiveness of the **Intervention** elements; either assay types or predator cue types. All articles considered for this analysis must have incorporated at least one of the **Comparator** elements and all of the **Outcome** elements listed in Table 1.

### Study validity assessment

To assess the risk of bias in each study, we evaluated the risk of biases from 6 criteria as defined in the CEE Critical Appraisal tool v.0.3 [64]: confounding biases; post-intervention/exposure selection biases; misclassified comparison biases; detection biases; outcome reporting biases; and outcome assessment biases. Factors identified by the review team and stakeholders that may confound the effectiveness of a behavioural assay or predator cue were scored during the meta-data extraction (e.g. species, sex, population origin, control type).

Using our Critical Appraisal checklist (adapted from the CEE Critical Appraisal tool; Additional file 3), we assessed the risk of bias for each of the aforementioned criteria (low, medium or high) and rated the overall risk of bias (low, medium or high) for each study. There were many cases where multiple studies (from the same article) were part of one experiment, for example, where each study considered a different treatment, or a different behaviour measured. In these instances, one checklist was completed, unless the review team were unable to evaluate them as one because different studies posed different risks of bias. In this case, and in cases where studies were from the same article but from a different experiment, independent checklists were completed. To evaluate the consistency of critical appraisal decisions, two members of the review team assessed an overlap of 20 studies (9.5%) and reached the same conclusion for each of them. In the statistical analysis, studies were weighted according to their risk of bias as described below (data synthesis).

### Data coding and extraction strategy

The variables detailed in Table 2 were extracted or scored from included studies where possible.

**Table 2:** Variables extracted from included studies

Category	Variable	Scoring options
Species	Common name	E.g. Cheetah
	Latin name	E.g. *Acinonyx jubatus*
	IUCN conservation status	E.g. Endangered
	IUCN threats to species	E.g. Invasive non-native/alien species/diseases
	Sex	Male, female, both
	Population Source	Captive, havened, wild
	Size	Small < 5 kg, medium 5–20 kg, or large > 20 kg
Assay	Assay type	E.g. flight initiation distance, trap behaviour, giving-up density
	Behaviour measured	E.g. avoidance, docility, exploratory behaviour, fear
Type of predator exposure	Comparison between populations with varying exposure to predators	Yes/no
	Use of predator cue	Yes/no
	Direct or contextual	
	Acoustic, visual, or olfactory	A, V, O, AV, AO, VO, or AVO
	Type of predator	E.g. terrestrial or aerial
Cue properties	Type of cue	E.g. faeces, urine, call, taxidermied model
	Did the cue move?	Yes/no
	Size of cue	Small < 5 kg, medium 5–20 kg, large > 20 kg
	Cue material	Organic or synthetic
Robustness of methods	Sample size	• Number of individuals • Number of populations (treatment groups) • Number of repeat measures per individual • Number of repeat measures per population
	Measure of repeatability	• Within individuals • Within populations
	What was the control	Blank, stimulus, or population
	If/how the methods were validated	E.g. fate of individuals, success criteria
	Effect size (difference in means between treatment and control group)	• Mean response (and standard deviation) of treatment group • Sample size of treatment group • Mean response (and standard deviation) of control group • Sample size of control group -OR- • Median of treatment group • Minimum and maximum of treatment group • Interquartile range of treatment group • Median of control group • Minimum and maximum of control group • Interquartile range of control group

For the quantitative component, we extracted the mean response of each treatment, its corresponding variance (standard deviation, standard error or confidence intervals), and the sample size for each treatment. Where articles alternatively present the median, minimum, maximum and interquartile range, we extracted these values, and used them to estimate the mean and standard error (please see below). In articles where such values were presented graphically, we measured the values directly from the figures (with the axes as scale bars) using the software *Image J* [65]. For a worked through example of effect size extraction using *Image J*, please see Addition file 6. Data were recorded using a customised data sheet (Additional file 5). To ensure consistency in data extraction, 20 studies (9.5%) were cross checked by two review-team members. Both members came to the same conclusions for all data extracted, and a pairwise t-test (in the R environment [59]) revealed no difference in the values extracted using *Image J* between scorers. We contacted authors from 17 articles to follow up missing information; we received four replies, and excluded the remaining 13 articles. Additional information regarding the species tested (threat status, threats faced, distribution) were collected from the IUCN Red List [66]. Extracted data can be found in Additional file 6.

### Potential effect modifiers/reasons for heterogeneity

The list of factors to be investigated by the review were compiled using the expertise of the review team, incorporating suggestions from stakeholders. We may have unintentionally excluded some useful data by only searching articles written in the English language. There may be a bias in the types of animals for which measures have been developed, for example, threatened or charismatic species. The type of predator cue used may substantially affect the outcome, as less effective cues may not be representative of an individuals’ response to a true predation event [64–67]. For the most robust quantification of behaviour, methodology should use repeat measures, incorporate measures of repeatability, and validate the assays, for example, by quantifying the fitness outcomes of various behavioural responses [68, 69]. With such a systematic review, we hope to highlight where biases may be occurring, and reveal areas where more robust methodology is needed to guide the development of behavioural assays.

### Data synthesis and presentation

The results from this systematic review are presented both as a narrative synthesis to address the primary question and as a quantitative analysis to address the secondary question [56]. To answer the primary question, *what behavioural assays have been used to quantify anti-predator responses in mammals*, we collated two tables of findings, and discuss specific examples and descriptive statistics in the text.

The secondary question, *which assay-types and predator cues elicit the greatest behavioural response,* has been answered via extracting meta-data. To make studies comparable, we converted all observations of effect sizes and their variance/error margins to means and standard deviation. If articles only provided standard errors these were transformed into standard deviation using the following equation:

$$\mathrm{SD}=\mathrm{SE}*\surd n$$

where $\mathrm{SE}$ is standard error, and $n$ is sample size. If articles only provided 95% confidence intervals, normal distribution was assumed, and these were transformed into standard deviation using the following equation:

$$\mathrm{SD}=\sqrt{n}\left(\frac{\mathrm{UL}-\mathrm{LL}}{3.92}\right)$$

where $n$ is sample size, and $\mathrm{UL}$ and $\mathrm{LL}$ are the upper and lower confidence limits respectively. There was one instance where an article provided mean deviation. From these values we estimated standard deviation by multiplying the values by a factor of $\surd \frac{2}{\pi }$. There was one article that provided the mean and the range. From these values, standard deviation was estimated by quartering the range ($\frac{1}{4}(Max-Min)$). If articles presented their results as box plots (providing the median, interquartile range, and minimum/maximum), we used the following equation [67] to estimate the mean:

$$\mu \approx \frac{a+2{q}_{1 }+2m+2{q}_{3 }+b}{8}$$

where $a$ is the minimum, ${q}_{1}$ and ${q}_{3}$ are the first and third quartiles respectively, $m$ is the median, and $b$ is the maximum. We estimated standard deviation following a method devised by Wan and colleagues [68]:

$$S\approx \frac{1}{2} \left(\frac{b-a}{\varepsilon \left(n\right)}+ \frac{{q}_{3 }-{q}_{1 }}{\upeta \left(n\right)}\right).$$

Both $\varepsilon$ and η are functions of $n$ as defined in [68] (Additional file 7). Using the treatment means, standard deviations and sample sizes extracted and estimated from each study, we then calculated a standardized measure of effect size for differences between means using Hedges’ g [63]:

$$g=\frac{{\mu }_{t - }{\mu }_{c }}{{S}_{p}}$$

where ${\mu }_{t}$ is the mean of the treatment group, ${\mu }_{c}$ is the mean of the control group and ${S}_{p}$ is the pooled standard deviation. The formula for pooled standard deviation is:

$${S}_{p}=\sqrt{\frac{\left({n}_{t}-1\right){s}_{t}^{2}+\left({n}_{c}-1\right){s}_{c}^{2}}{\left({n}_{t}-1\right)+ \left({n}_{c}-1\right)}}$$

where ${n}_{t}$ and ${s}_{t}$ are the number of observations and standard deviation for the treatment group respectively, and ${n}_{c}$ and ${s}_{c}$ are the number of observations and standard deviation for the control group respectively. Hedges’ g was chosen over other effect size measures such as Cohen’s d, as it is suited to a range of sample sizes and because it facilitates comparisons across studies by weighting each measure based on the number of observations [69]. As we were interested in the magnitude of difference in effect sizes between the treatment and control conditions, rather than the direction of difference, we used absolute values of Hedges’ g in our analysis. Finally, to evaluate potential publication bias, we created and visually inspected a funnel plot (using the metafor package [70] in R [59]) which suggests an approximately symmetrical distribution of studies (Additional file 8).

We built two mixed effects models using R [59] to identify which predator cue types and behavioural assay types elicit the greatest difference in effect size (Hedges’ g), while controlling for potential confounding factors where possible. We included each article’s unique identifier as a random effect in both models to account for the non-independence of multiple effect sizes from each article. We also included genus as a random effect to account for potential bias towards taxa more heavily represented in the literature (e.g., *Rattus*). Studies were weighted based on their risk of bias determined through critical appraisal whereby studies with a low, moderate, or high risk were weighted with a value of 3, 2, or 1 respectively.

Model selection was used to determine factors that most influenced the response variables of each model. To select the best model, we used likelihood ratio tests to test the relative weight of each variable by comparing a model with that parameter removed to the full model [71]. We also compared AIC values of candidate models to ensure the final models were the most parsimonious (∆AIC < 2) [72]. As we did not have observations for all combinations of categories, we were unable to test for the following biologically relevant interaction effects from the assay type model: assay type and species type; behaviour measured and species size; assay type and predator type (aerial/terrestrial); source population (captive, wild, haven) and predator source (native, novel, historic, introduced); behaviour measured and sex. From the predator cue type model, we were unable to test for: cue type (direct/indirect) and cue structure (synthetic/organic); cue type and source population. In the predator cue type model, the cue properties variable (the appropriate combination of visual, auditory and olfactory) showed high collinearity and was excluded from the analysis.

## Review findings

### Review descriptive statistics

**Fig. 3:**
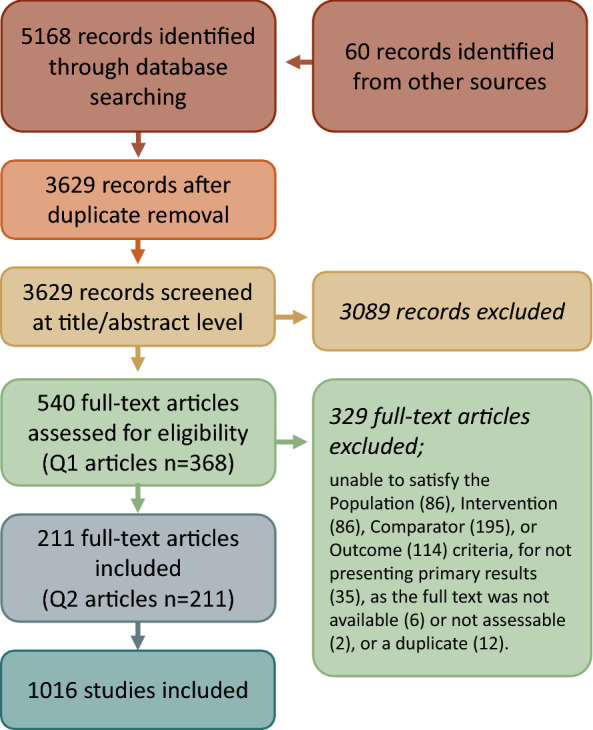
Flow diagram detailing articles and studies identified and excluded at each step of the search process

Our search returned 5228 articles (18 theses + 5210 peer reviewed articles; totalling 3629 after duplicates had been removed) that had publication dates between 1984 and 2022. Of these, 211 articles passed the screening process, resulting in 1016 studies (Fig. 3). Full lists of search results by source, eligible studies, and reasons for exclusion, are available in Additional file 9. Critical appraisal determined that all studies posed a low risk of bias. Critical appraisal checklists for each study can be found in CADIMA (please see data availability section).

### Species assayed

**Fig. 4:**
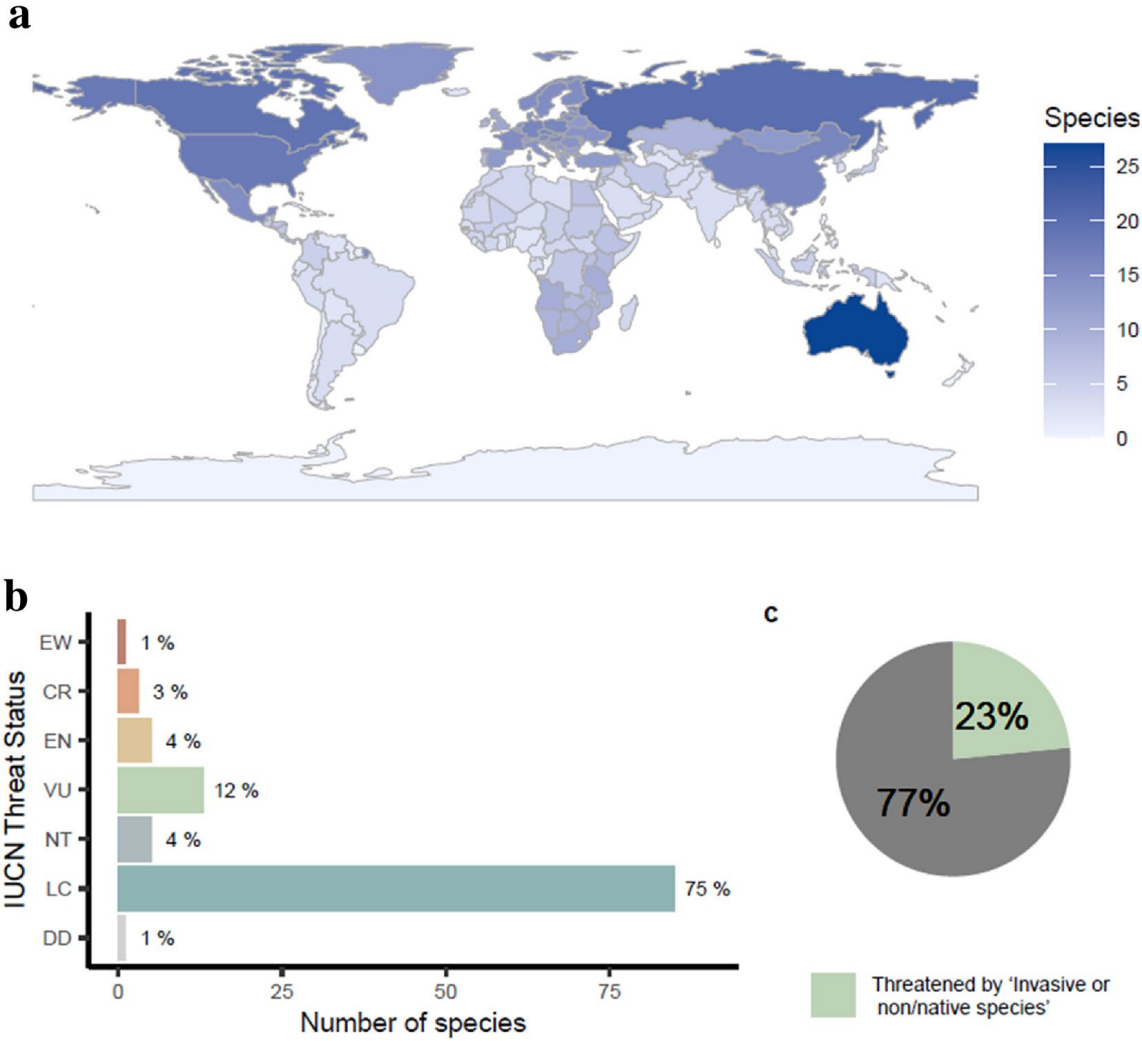
Species for whom anti-predator behaviours have been assayed: **a** Number of species assayed per country that they are found in, **b** species extinction risk category according to the IUCN Red List (DD: Data Deficient, LC: Least Concern, NT: Near Threatened, VU: Vulnerable, EN: Endangered, CR: Critically Endangered, EW: Extinct in the Wild), and **c** proportion of species threatened by ‘Invasive or non/native species’

We identified 126 species for whom anti-predator responses have been investigated. Australia contained the most assayed species (27 species), followed closely by species from North American and Asian continents (18 and 22 species respectively). Species from South America and Africa were underrepresented in the literature (Fig. 4a). Excluding domesticated species, the threat status of all 126 species has been assessed by the International Union for the Conservation of Nature (the IUCN). Most species were listed as Least Concern, with only 20% of assayed species listed as threatened (Vulnerable, Endangered, Critically Endangered), or Extinct in the Wild; Fig. 4b. According to the species’ Red List profiles, one quarter are threatened by “Invasive non-native/alien species” (Fig. 4c).

### Range of behavioural assays

From the literature, we identified six major types of behavioural assays: stimulus presentations (71.3% of studies), feeding station (14.6%), behavioural focals (3.7%), giving-up density (7.4%), capture probability (2.0%), and flight initiation distance (1.0%). These assays measured five primary behaviours: activity, escape, exploration, foraging and vigilance (Table 3). Additional behaviours that did not fall into these categories, such as aggression or grooming, were rarely measured, and were grouped into an “other” category.

**Table 3:** Behavioural assays used to quantify anti-predator responses in mammals

Assay	Description	Behavior measured	Application references
Behavioural focal	Ad libitum observations of unprovoked behaviours, without the presentation of a stimulus	Activity	[5, 73-76]
		Exploration	[5]
		Foraging	[77, 78]
		Vigilance	[74, 77, 78]
		Other	[74]
Capture probability	The probability of an animal entering a trap. Traps may be treated with predator cues	Exploration	[79-86]
Feeding station	Monitoring of behaviour around a food source. May include a stimulus	Activity	[51, 87-96]
		Escape	[97, 98]
		Exploration	[15, 51, 89-91, 93, 95, 97, 99-103]
		Foraging	[15, 51, 87-89, 91, 92, 94, 95, 100, 102, 104-116]
		Vigilance	[15, 87, 89-91, 95, 97, 101, 105, 117, 118]
Flight initiation distance	The distance at which the animal flees from an approaching predator stimulus	Escape	[15, 119-124]
Giving-up density	The food density at which the animal chooses to leave the foraging patch. May include a stimulus	Foraging	[75, 79, 85, 91, 92, 118, 125-159]
Stimulus presentation	Behavioural observations of an animal following the presentation of a predator or other stimulus	Activity	[41, 45, 160-234]
		Escape	[15, 49, 52, 121, 163, 166, 186, 187, 198, 199, 201-204, 219, 229, 232, 235-248]
		Exploration	[52, 102, 120, 153, 161, 165, 167, 172, 174, 176-178, 180, 184, 185, 189, 190, 194, 195, 198, 199, 201-203, 205-207, 210-213, 217, 219, 222, 225, 229, 230, 233, 237, 240, 241, 244, 247, 249-262]
		Foraging	[40, 41, 102, 161-164, 167, 171, 179, 185, 198, 211, 230, 234, 237, 247, 263-271]
		Vigilance	[41, 52, 102, 122, 160-166, 170-172, 174-176, 178, 182, 192, 198, 201-204, 217, 231, 236, 237, 239, 247-249, 251, 258, 263-267, 271-283]
		Other	[161, 201, 203, 204, 217, 225-227, 229, 233, 258, 262, 277, 284]

*Behavioural focals* were commonly used to compare anti-predator responses in populations with varying predator exposure or naïveté, without the use of a predator stimulus. Ross and colleagues [5], for example, used this method to compare anti-predator responses between two populations of greater bilbies (*Macrotis lagotis*): a naïve havened population and a population exposed to cats (an introduced meso-predator). This method facilitates observation of general behaviours, such as foraging and vigilance trade-offs, and can be used to discern varying personality phenotypes (e.g., bold or shy individuals) and to evaluate neophobia (aversion to novelty [285]).

*Capture probability*, which represents an animal’s exploratory behaviour, is an innovative way to assay behaviour using live animal traps. This method was predominantly used to assay behaviour in small rodents. For example, both Russell and colleagues [83] and Dickman and colleagues [86] evaluated anti-predator responses in rodents, including Australian bush rats (*Rattus fuscipes*), wood mice (*Apodemus sylvaticus*) and shrews (*Sorex araneus*), by setting an array of scented traps—treated with predator odours or not—giving the focal species a choice of trap to explore. This particular assay can discern whether individuals recognise the scent of predators, and behave differently when there is immediate evidence of predators.

*Feeding stations* can be used to reveal how animals forage; a behaviour that commonly trades off against anti-predator behaviours such as vigilance. Feeding may make an individual vulnerable, and how they allocate their time to vigilance versus other behaviours can reveal the risk they perceive. For example, Saxon-Mills and colleagues [51] compared the foraging, exploratory and activity behaviours of burrowing bettongs (*Bettongia lesueur*) from both naïve and cat exposed populations at predator scented food trays. Such methodology provides insight into an animal’s priorities, and can highlight contexts of interest—for example, an animal ignoring predator cues in the pursuit of food may be experiencing resource limitation.

*Flight initiation distances* are a particularly cost-effective method for quantifying anti-predator responses because they can be conducted with very little, or completely without specialized equipment. Cappa and colleagues [119], for example, used this method to identify the predation risk perceived by guanacos *(Lama guanicoe)* to the presence of human poachers.

*Giving-up densities (GUD)* are an effective tool for quantifying perceived risk. General foraging patterns and perceived risk between populations can be compared, or else can be paired with a predator stimulus to reveal predator awareness. GUD’s can also be used to establish landscapes of fear. Makin and colleagues [118], for example, used this method to evaluate the predation risk perceived by kudu (*Tragelaphus strepsiceros*), sable (*Hippotragus niger*) and warthogs (*Phacochoerus africanus*) before and after the release of a predator species, the African wild dog (*Lycaon pictus*). Using a slightly different approach, Jolly and colleagues [143] used giving-up densities to quantify the ability of Northern quolls (*Dasyurus hallucatus*) to detect and identify predators by measuring the food left from a choice of three GUD trays, each scented with a different predator odour (including a control).

*Stimulus presentations* represent a broad array of assays that facilitate investigation into a wide range of behaviours. For example, Aschemeier and colleagues [231] examined how woodchucks (*Marmota monax*) responded to conspecific and heterospecific alarm call playbacks. Shier and colleagues [175] compared the activity and vigilance behaviour of naïve and trained prairie dogs (*Cynomys ludovicianus*) confronted with live predators: ferrets, hawks, and snakes. Blumstein and colleagues [163] observed activity, foraging, vigilance, and escape behaviours of yellow-bellied marmots (*Marmota flaviventris*) presented with life-sized predator photographs.

### Range of predator cues

**Fig. 5:**
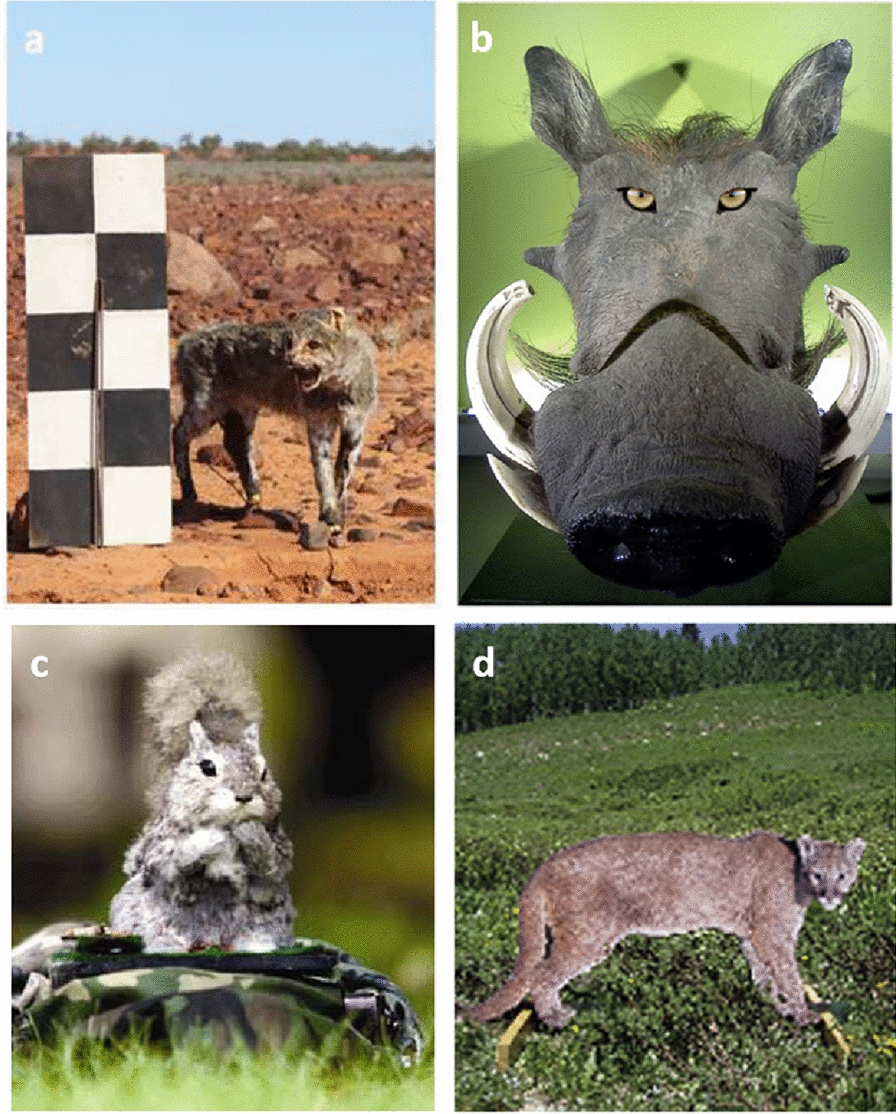
Examples of visual predator cues used to quantify anti-predator responses in mammals; taxidermied cat (**a**), warthog modified to include predator eyes (**b**), mechanical gray squirrel robot (**c**), and life sized photograph of mountain lion (**d**) (Adapted from [52, 100, 163, 279] respectively)

Predator cues fell into two broad categories: direct cues, or contextual cues. Direct predator cues indicate that a predator is immediately present, whereas contextual cues imply that the predator may have recently been present, or is nearby. Within these categories, predator cues can target different sensory systems, for example, cues may include visual (e.g. Fig. 5), olfactory, and/or audible stimuli. We provide examples of these categories of stimuli, and list some of their applications in Table 4.

**Table 4:** Types of predator cues used to quantify anti-predator responses in mammals, and examples of applications in studies

	Type	Example	Application
Direct	Visual	Predator model (taxidermied, artificial, photograph)	[52, 97, 163]
	Audible	Predator call	[89, 164, 266]
	Olfactory	Predator body odour	[90, 171]
	Visual + Olfactory	Predator fur/skin/feathers	[88]
	Visual + Audible + Olfactory	Live predator	[125, 175, 238]
Contextual	Visual	Conspecific or heterospecific alarm behaviour	[279]
	Audible	Conspecific or heterospecific alarm call	[263, 280]
	Olfactory	Predator urine, predator scent mark	[253, 286]
	Visual + Olfactory	Predator faeces	[237, 251]
	Visual + Olfactory/Audible	Injured conspecific	[102]

### Repeatability and assay validation

Measuring the repeatability of behavioural assays allows observers to determine whether responses are consistent across time and/or across contexts. In the case of anti-predator responses, we would expect behavioural assays to be highly repeatable, and for individuals to respond consistently to potential predation scenarios. Of studies that had repeat observations of known individuals, 15.4% calculated the within individual repeatability of the behaviours measured. Another robust approach is to validate the methodology, for example, by determining how the quantified responses correlate to survival, or to successful predator escape. While this is not always feasible, across all studies, only 2.4% validated their behavioural assays.

### Most effective behavioural assays

**Fig. 6:**
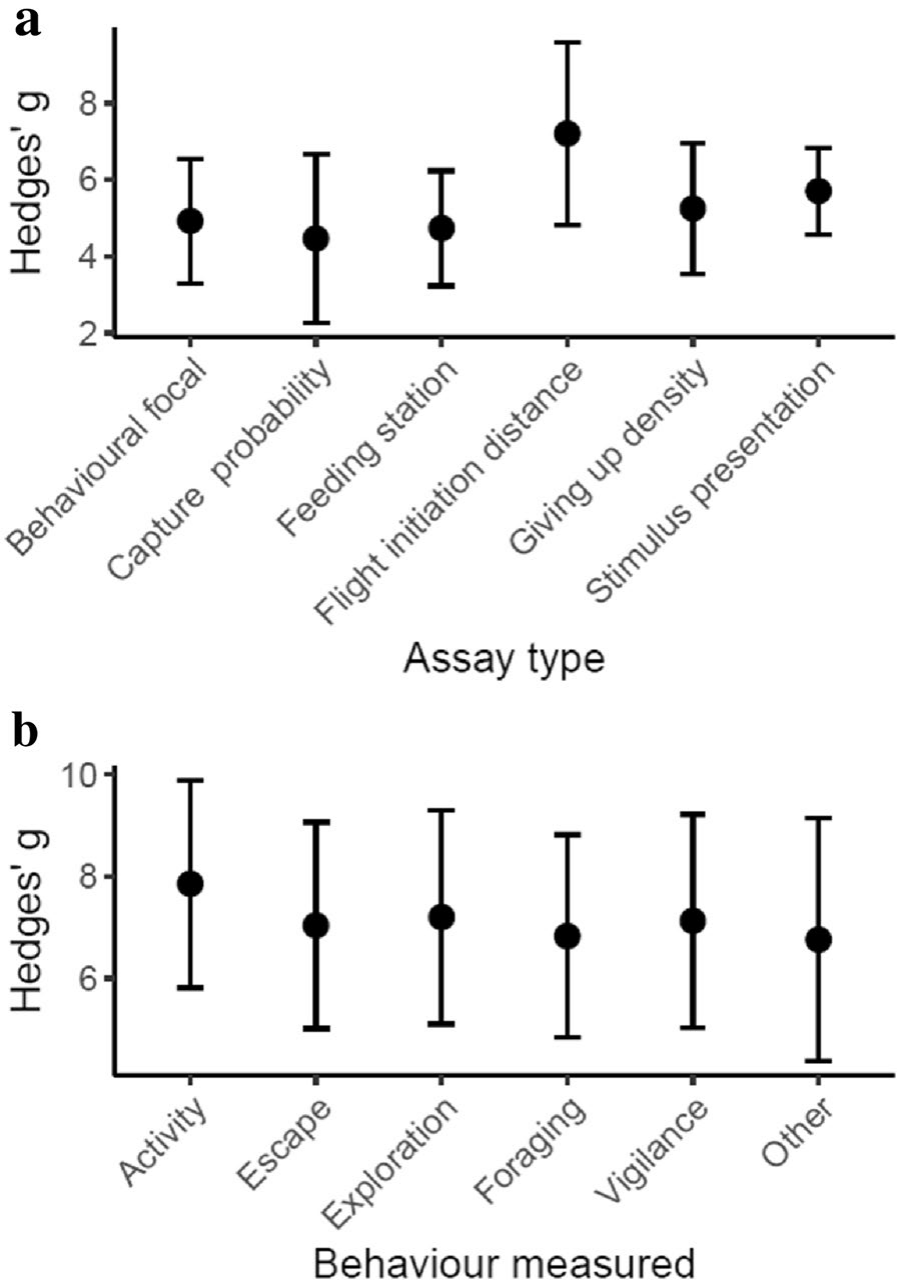
Effect of (**a)** assay type and (**b)** behaviour measured on differences in effect size, Hedges’ g. Error bars indicate mean +− standard errors of the mean.

Across studies (n = 973), there was no one behavioural assay type that elicited greater effect sizes (Hedges’ g) than others (Table 5). Model predictions show that studies utilizing flight initiation distances as a metric for anti-predator responses had slightly higher Hedges’ g (Fig. 6a), but this difference was not statistically significant. Similarly, there was no one behaviour measured that lead to greater effect sizes (Table 5). Model estimates show that studies measuring activity had slightly higher Hedges’ g scores (Fig. 6b). Studies of small (< 5 kg) and medium (5–20 kg) species, had lower Hedges’ g compared to studies of large (> 20 kg) species (Table 5). Studies that analysed sex independently reported higher Hedges’ g than those that pooled responses from males and females (Table 5). In contrast, population source (captive, wild or haven), predator type (aerial or terrestrial), and predator source (historic, native, novel, or introduced) did not influence Hedges’ g scores, and each of these covariates was excluded during model selection.

**Table 5:** Influence* of behavioural assay properties on effect sizes (Hedges’ g)

Fixed effect	Slope	S.E	df	p-value
Intercept	2.755	1.799	–	–
AssayType_CaptureProbability	− 0.459	2.131	5	0.754
AssayType_FeedingStation	− 0.188	1.647		
AssayType_FlightInitiationDistance	2.278	2.435		
AssayType_GivingUpDensity	0.325	1.786		
AssayType_StimulusPresentation	0.781	1.542		
BehaviourMeasured_Other	− 0.070	0.995	5	0.431
BehaviourMeasured_Activity	1.029	0.625		
BehaviourMeasured_Escape	0.209	0.787		
BehaviourMeasured_Exploration	0.373	0.638		
BehaviourMeasured_Vigilance	0.298	0.628		
SingleSex	4.406	0.704	1	< 0.001
Size_Medium	− 2.113	1.196	2	0.071
Size_Small	− 2.198	0.995		

*The slope, standard error (S.E), and degrees of freedom (df) for each variable in the final model is reported (with levels of a particular variable indicated after the underscore), as is the p-value resulting from log likelihood tests of the final model (SingleSex + Size) with and without each respective variable

### Most effective predator cues

Of the studies that incorporated predator cues (n = 867), the properties examined here did not substantially explain effect sizes. Neither cue structure (organic or synthetic), cue movement (yes or no), cue type (direct or indirect), predator type (aerial or terrestrial) or population source (wild, captive, havened) had any effect and all were eliminated during model selection. Predator source (historic, introduced, novel, or native) was not excluded during model selection but did not have a significant effect (Table 6.) Control type, however, had a substantial effect on Hedges’ g: studies incorporating blank control types (i.e. lack of stimulus) had larger effect sizes than those that utilised control stimulus cues. As with the assay model above, studies that analysed males and females independently had higher effect sizes (Table 6).

**Table 6:** Influence* of predator cue properties on effect sizes (Hedges’ g)

Fixed effect	Slope	S.E	df	p-value
Intercept	4.244	0.954	–	–
CueSize_M	− 0.537	0.671	2	0.394
CueSize_S	− 1.033	0.764		
ControlType_Stimulus	− 1.890	0.675	1	0.006
SingleSex	4.177	0.733	1	< 0.001
PredatorSource_Introduced	− 0.013	0.799	3	0.592
PredatorSource_Native	− 0.647	0.610		
PredatorSource_Novel	− 0.904	1.370		

*The slope, standard error (S.E), and degrees of freedom (df) for each variable in the final model is reported (with levels of a particular variable indicated after the underscore), as is the p-value resulting from log likelihood tests of the final model (ControlType + SingleSex) with and without each respective variable

## Potential confounding factors

### Anti-predator responses or responses to novel stimuli?

Creating realistic predator cues is a challenge. While studies aiming to quantify anti-predator responses may find differences among individual responses to predator cues, it is difficult to discern whether these responses accurately reflect how individuals would act in the face of real predators. Observed differences may instead be a response to a novel environment or stimulus (e.g. neophobia [285]), or may relate more to an animals’ personality (repeatable inter-individual differences in behaviour [23]) than their ability to evade predation. It is crucial to be able to distinguish between these scenarios, especially in the context of conservation translocations, where mistaking a curious response to a predator stimulus for predator recognition and avoidance abilities may result in high mortality if such naïve individuals are exposed to predators.

**Fig. 7:**
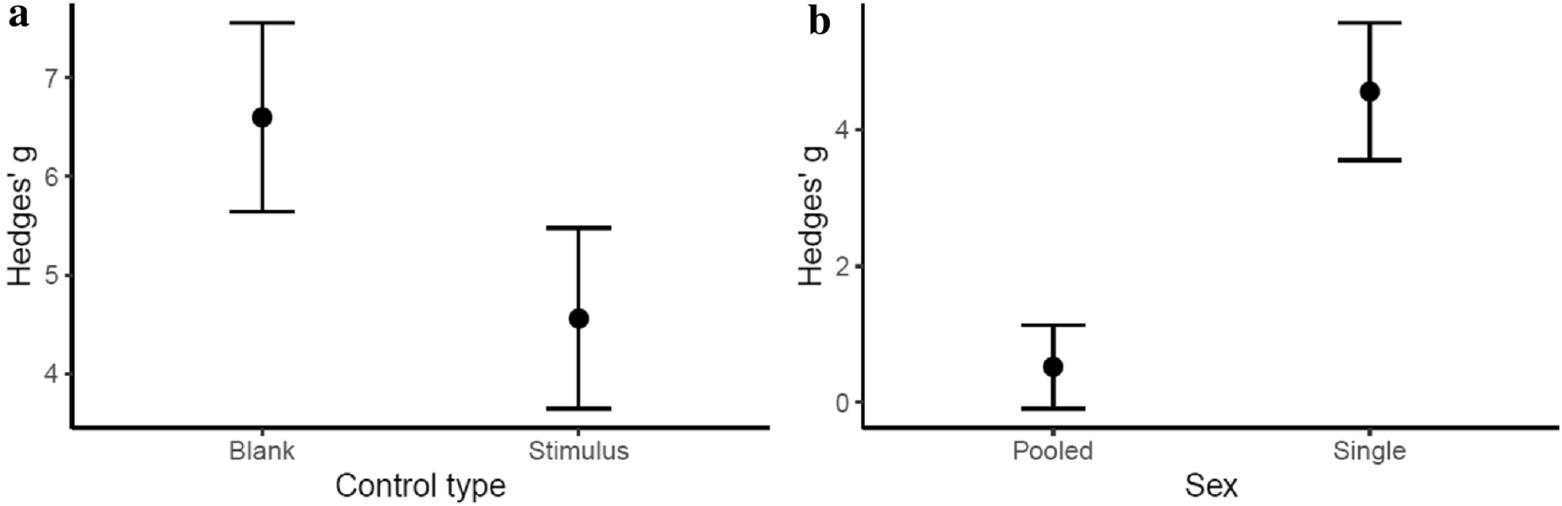
Difference in effect sizes between studies that had non-predator stimulus control cues (stimulus), compared to those that had the absence of any stimuli as a control (blank) (**a**), and between studies that pool males and females, compared to those who analyse males and females independently (**b**). Error bars indicate mean +− standard errors of the mean

We found that studies that used blank controls compared to those that used non-predator stimulus controls had significantly larger effect sizes (p < 0.01; Fig 7a). To avoid the confounding effect of novel stimuli, it is essential to use appropriate control cues. For example, it could be appropriate to use a predator faeces as a stimulus, and herbivore faeces as a control, or a predator vocalisation stimulus and a song from a non-threatening bird as a control. Of those studies that incorporated predator cues, most used suitable controls, however, 21% used “blank” controls (i.e. the absence of any stimulus as a control). While this is suitable when comparing responses at different levels of predator exposure, it is not advisable when predator cues are used. This is because without an appropriate control, it cannot be discerned whether the behaviours measured are in response to the cue of a predator, or merely those associated with investigating a novel or unusual cue. Hence, to avoid incorrect over-inflation of effect sizes, studies using predator cues should select their control stimuli carefully.

### Single or pooled sexes

Owing to differences in reproductive strategies, males and females may employ different anti-predator responses. For example, males, who might regularly traverse unfamiliar territories in pursuit of mating opportunities, may freeze when confronted with a predator as they would be unfamiliar with refuges in the area. In contrast a female, who remains within her known territory, may flee in the face of a predator as she may be aware of refuges, and may aim to distract the predator away from vulnerable young. For instance, this difference has been observed in rats where males respond to fear with a “freeze” response, whereas females prefer the “dart” response [287]. Without differentiating between such strategies, it might appear that some individuals display inappropriate anti-predator responses (e.g., freezing when they are expected to flee). Failing to account for sexual dimorphism in behavioural responses may thus cause anti-predator abilities to be underestimated. Our results showed that effect sizes from studies that analysed males and females independently were approximately three times greater than those who analysed sexes in combination (Table 5). While it is not always possible to account for sex, for example, in studies of wild animals where sex is not known, this finding highlights the important effect that behavioural sexual dimorphism might have on studies of animal behaviour.

### Organic and synthetic predator cues

Thirteen percent of studies reviewed here used artificial or synthetic predator cues, for example, plastic predator models, or synthesized volatile components of faeces (trimethylthiazoline, TMT; fox odour). We found no differences in effect sizes for organic or synthetic predator cues (Table 6). A selection of studies that we came across, however, reported that some synthetic compounds, such as TMT, were less effective at eliciting responses than organic alternatives [182, 185, 210, 212, 244]. While our findings did not support this, we only compared organic and synthetic cues broadly, and were unable to draw these comparisons among cues with different properties (olfactory, visual, auditory). We recommend that as a precaution, organic cues be selected over synthetic ones where possible, especially in the case of olfactory cues.

### Review limitations

Our review reveals a lack of studies on species from South America and Africa (Fig. 4a). This finding could be explained by the fact that we only considered articles written in English. Despite a search strategy that also targeted grey-literature, the returned results were almost exclusively peer-reviewed articles (8% theses, 92% peer-reviewed journal articles, 0 articles from government or NGO reports). Further, of studies that reported means and error margins (rather than box plots), 14.9% did not specify what unit the error margin represented (i.e. standard deviation, standard error, or confidence intervals), which, despite attempts to contact corresponding authors, forced us to reject some studies from the quantitative analyses. During the article screening process, we may have unintentionally introduced biases by only performing consistency checks on a subset of the articles, however, given our high inter-rater reliability scores throughout, we are confident that any such biases would have minimal effect on our findings. Finally, our analysis was limited by our inability to examine interaction effects between variables, for example, the interaction between cue type and cue properties (olfactory, visual, auditory). Behavioural responses are complex, and without investigating fine scale details, such as the effectiveness of predator cues that target different sensory systems among taxa, our ability to comment on the most effective methodology (behavioural assays and predator cues) was hampered. Our review delivers a detailed global overview of methodology for quantifying anti-predator responses in mammals, but owing to limitations in our data set, was unable to provide taxa or sensory system specific insights.

## Review conclusions

### Implications for management

This review identifies six major types of behavioural assay (behavioural focals, capture probability, feeding station, flight initiation distance, giving-up density, and stimulus presentations) that measure five primary behaviours (activity, escape, exploration, foraging and vigilance). As quantified by differences in effect sizes, there was no behavioural assay, nor particular behaviour that was universally the best at capturing variation in anti-predator responses. Similarly, there were no predator cue types or properties found to be universally most effective.

Population managers seeking to quantify anti-predator responses can use this review to understand the primary assay types and behaviours that have been recorded in past research. Population managers can also find information to replicate existing assays for a particular species, with references to specific examples, in Additional file 9. Our review has also highlighted the importance of controlling for potentially confounding factors. We advise managers to take care in selecting appropriate control stimuli (where necessary), to consider using organic predator cues over synthetic ones, and to be alert to the possibility of sexual dimorphism in anti-predator behaviour.

Despite specifically targeting government and NGO sources during our search strategy, no studies were returned. This suggests that despite growing recognition of the impact of anti-predator behaviour on the persistence of mammals [288], measurement of anti-predator behaviours are not being widely incorporated into conservation monitoring and management. Given that behaviour is a key trait mediating impact from predation, we urge population managers to incorporate an understanding of these behaviours into future management strategies.

### Implications for research

Information provided by anti-predator behavioural assays can be critically important for informing species conservation and management. To further this analysis, future work could aim to quantify how well different behavioural assays can predict vulnerability to predation in mammals. Future research might also be directed at categories that were underrepresented in this review, such as species from South America or Africa, and species highly threatened with extinction. Additionally, further investigations should focus on understanding the anti-predator responses of mammals impacted by invasive species, or species that largely exist in isolation from predators (e.g., in captive breeding programs, or in predator-free havens). An enhanced understanding of anti-predator responses would be most beneficial for the conservation management of such species.

Gaps in our data set limited our ability to investigate the most effective behavioural assays and predator cues by preventing us from examining interaction effects. It is likely that such effects would provide valuable insights into complex behavioural responses. Future research could investigate these intricacies within a particular group of mammals, or more broadly, in another taxonomic group. The search strategy developed here may be applied to other systematic reviews and used for such a purpose. As we used a broad search strategy independent of species information, and scored the satisfaction of each article against all criteria, parties interested in replicating this review for alternate taxonomic groups (e.g. fish, reptiles, amphibians, birds) could refer to Additional file 4 to extract articles from our search which satisfy the I, C, O criteria, but not our Population criteria, and re-assess this subset against a new Population criteria.

We encourage future studies of anti-predator responses to incorporate measures of individual repeatability, as it not only strengthens behavioural inferences that can be drawn, but also allows us to assess the robustness of the particular behavioural assay. Assays of repeatable behaviours can facilitate robust comparisons among individuals, and between populations, offering valuable opportunities to compare anti-predator abilities between naïve and predator-exposed populations. We further encourage studies of this nature to consider validating their methodology, increasing both the reliability of their results, and the robustness of their behavioural assays, which may then be adopted by future studies. Finally, we urge future studies of this nature to be detailed in their reporting of statistical analyses and findings (including units of error reported) to ensure accurate, thorough, and transparent communication of results and to facilitate subsequent meta-analysis.

## Supplementary Information

**Additional file 1.** ROSES checklist.

**Additional file 2.** List of benchmark and test scoping articles.

**Additional file 3.** CEE critical appraisal tool sheet.

**Additional file 4.** Exclusion list (reasons for exclusions).

**Additional file 5.** Customised data collection sheet.

**Additional file 6.** Guide to using Image J for data extraction.

**Additional file 7.** Definition of equation functions.

**Additional file 8.** Funnel plot to assess publication bias.

**Additional file 9.** Data extraction results.

## Data Availability

All data from this review are available in CADIMA (https://www.cadima.info/index.php/area/evidenceSynthesisDatabase#:~:text=Identifying%20the%20most%20effective%20behavioural%20assays%20and%20predator%20cues%20for%20quantifying%20anti%2Dpredator%20responses%20in%20mammals).
