# Supplementary material for: Identifying the most effective behavioural assays and predator cues for quantifying anti-predator responses in mammals: a systematic review
Source: Environ Evid. 2023 Apr 1;12:5. doi: 10.1186/s13750-023-00299-x (PMC11378833; doi:10.1186/s13750-023-00299-x)
Supplement: Supplementary file 2 — Additional file 2. List of benchmark and test scoping articles. [file 13750_2023_299_MOESM2_ESM.docx]

SCOPING BENCHMARK ARTICLES

1. Blumstein et al., (2004). A Test of the Multi-Predator Hypothesis: Rapid Loss of Antipredator Behavior after 130 years of Isolation. Ethology 110, pp919—934
2. Hollings et al., (2015). Relaxation of risk-sensitive behaviour of prey following disease-induced decline of an apex predator, the Tasmanian devil. Proceedings: Biological Sciences, 282(1810)
3. Jones et al., 2004. Is anti-predator behaviour in Tasmanian eastern quolls (Dasyurus viverrinus) effective against introduced predators? *Animal Conservation* 7, pp155–160
4. Orrock, J.L. (2010). When the Ghost of Predation has Passed: Do Rodents from Islands with and without Fox Predators Exhibit Aversion to Fox Cues? *Ethology* 116, pp338–345
5. Ross et al., 2019. Reversing the effects of evolutionary prey naivete through controlled predator exposure. *Journal of Applied Ecology* 56, pp1761-1769
6. Steindler, L.A. et al., (2020). Exposure to a novel predator induces visual predator recognition by naïve prey. *Behavioural Ecology and Sociobiology* 74(102)
7. Steindler, L.A. et al., (2018). Discrimination of introduced predators by ontogenetically naïve prey scales with duration of shared evolutionary history. *Animal Behaviour* 137, pp133-139
8. Saxon-Mills, E.C. et al., (2018). Prey naivete and the anti-predator responses of a vulnerable marsupial prey to known and novel predators. *Behavioural Ecology and Sociobiology* 72(151)
9. Tay, N.E. et al., (2019). Predator exposure enhances the escape behaviour of a small marsupial, the burrowing bettong. *Animal Behaviour* 175, pp45-56
10. West, R. et al., (2017). Predator exposure improves anti-predator responses in a threatened mammal. *Journal of Applied Ecology* 55, pp147-156

INDEPENDENT SEARCH COMPREHENSIVENESS TEST ARTICLES

1. Bytheway & Banks, (2019). Overcoming prey naivete: Free-living marsupials develop recognition and effective behavioral responses to alien predators in Australia. *Global Change Biology* 25(5), pp1685-1695
2. Jolly et al., 2021. Trophic cascade driven by behavioral fine-tuning as naıve prey rapidly adjust to a novel predator. *Ecology*, 102(7),
3. Mella et al., 2010. Predator odour does not influence trappability of southern brown bandicoots (Isoodon obesulus) and common brushtail possums (Trichosurus vulpecula). *Australian Journal of Zoology*, 58, 267-272
4. Moseby et al., 2018. Designer prey: Can controlled predation accelerate selection for antipredator traits in naïve populations? *Biological Conservation* 217, pp213–221
5. Pickett et al., 2005. The influence of predation risk on foraging behaviour of brushtail possums in Australian woodlands. *Wildlife Research* 32, pp121-130
