# Supplementary material for: Identifying the most effective behavioural assays and predator cues for quantifying anti-predator responses in mammals: a systematic review
Source: Environ Evid. 2023 Apr 1;12:5. doi: 10.1186/s13750-023-00299-x (PMC11378833; doi:10.1186/s13750-023-00299-x)
Supplement: Supplementary file 6 — Additional file 6. Guide to using Image J for data extraction. [file 13750_2023_299_MOESM6_ESM.pdf]

Image J guide for Systematic Review Data Extraction

1. **The image.**

Take a screen shot (windows, shift, S) of the figure you are interested in (make sure it includes the scale/legend). Open the ImageJ program, and File -> Open, select this screenshot.

2. **Set the scale.**

Click on the “Straight segment” tool.

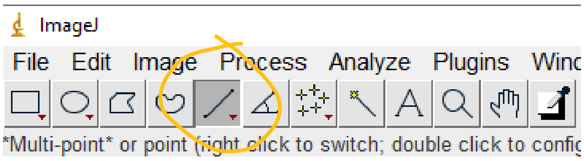

Zoom in so that you have a clear view of the axis of the figure (Ctrl + is the short cut). Then hold shift (which forces the line to be either vertical or horizontal) and click and hold the bottom (0 in this example below), and drag the mouse to the next tick on the axis (one tick is enough as the scale will be the same no matter the length that you measure). Release your mouse click, the yellow line should remain there.

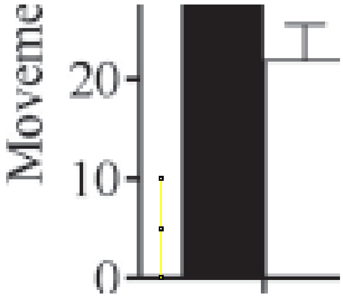

If you are happy with this line (it is exactly lining up with the two axis ticks) then click “Analyze” -> “Set Scale” and this box will pop up.

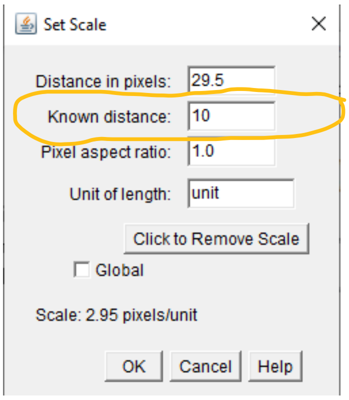

The distance in pixels is the length of the line relative to the image (don’t change this). You want to specify the *Known distance*. In the example above, this distance is 10. Units are not important here, as they will vary between every study so you can leave this as “unit”. Click OK.

3. Measuring the distances.

Using this same line tool as above, and again holding the shift key, draw a line from the base to the top of the bar that you want to measure.

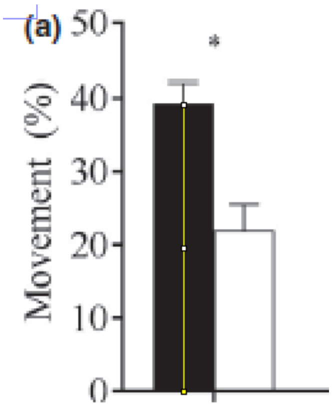

Then click “Analyze” -> “Measure”, or Ctrl M.

Results

File Edit Font Results

	Area	Mean	Min	Max	Angle	Length
1	12.058	32.684	32.667	33.667	90	37.077

This gives you the measurements in “units” compared to the scale that we specified, in this case we are interested in length. Then transfer this measurement to the excel sheet, reporting to 3 decimal places.
