# Supplementary material for: Identifying the most effective behavioural assays and predator cues for quantifying anti-predator responses in mammals: a systematic review
Source: Environ Evid. 2023 Apr 1;12:5. doi: 10.1186/s13750-023-00299-x (PMC11378833; doi:10.1186/s13750-023-00299-x)
Supplement: Supplementary file 8 — Additional file 8. Funnel plot to assess publication bias. [file 13750_2023_299_MOESM8_ESM.docx]

**Additional file 8**


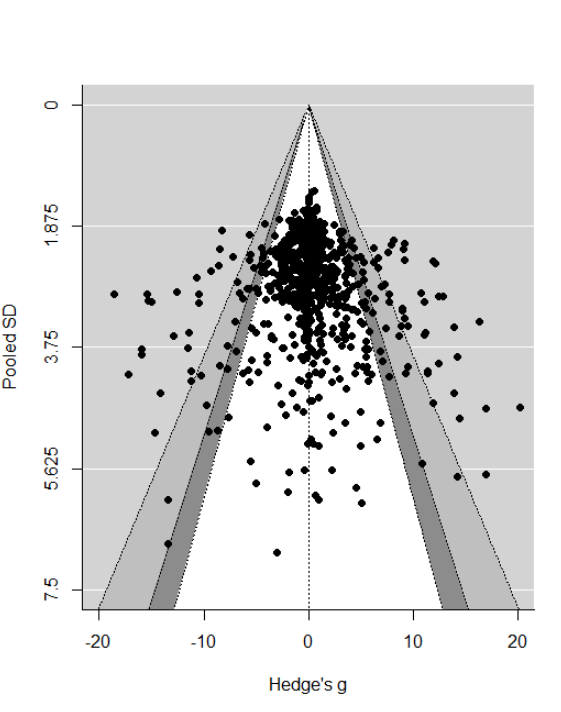


***Figure 1.*** Funnel plot to assess publication bias. The colours signify the significance level into which the effects size of each study falls (white = n.s., dark grey = p < .05, medium grey p < .01, and light grey p < .001)
